# Sequential Immune-Mediated Extrahepatic Complications of Hepatitis E Virus: A Case Report

**DOI:** 10.7759/cureus.113617

**Published:** 2026-07-29

**Authors:** Krishna Padarabinda Tripathy, Subhashree Mishra, Virani A Laxman, Palle Sree Hima Varsha, Satya Bharadwaj

**Affiliations:** 1 General Medicine, Kalinga Institute of Medical Sciences, Bhubaneswar, IND; 2 Internal Medicine, Kalinga Institute of Medical Sciences, Bhubaneswar, IND; 3 Medicine, Kalinga Institute of Medical Sciences, Bhubaneswar, IND

**Keywords:** autoimmune haemolytic anaemia, cytokine storm, extrahepatic manifestations, guillain–barré syndrome, haemophagocytic lymphohistiocytosis, hepatitis e virus, immune dysregulation, multisystem involvement, neuroimmunology, rare complications of hepatitis e

## Abstract

Hepatitis E virus (HEV) infection is classically described as causing self-limiting acute hepatitis. However, it is increasingly being recognised for a wide spectrum of immune-mediated extrahepatic complications involving the haematological, neurological, renal and pancreatic systems. These manifestations are more commonly seen as isolated presentations in individual patients. We report a 59-year-old man with serologically confirmed acute hepatitis E, in whom alternative infectious, autoimmune, and malignant etiologies were excluded, who developed three distinct immune-mediated complications in temporal sequence during a single hospital admission: autoimmune haemolytic anaemia (AIHA) at presentation, secondary haemophagocytic lymphohistiocytosis (HLH) within one week, and Guillain-Barré syndrome (GBS) on day 17. The chronological evolution of these complications suggested a progressive triphasic immune-mediated response following acute HEV infection. Each complication was diagnosed using established criteria and managed with targeted therapy-corticosteroids and supportive care for AIHA; etoposide, escalated dexamethasone, and cyclosporin for HLH; and intravenous immunoglobulin for GBS. The sequential development from early autoantibody-mediated haemolysis to hyperinflammatory macrophage activation and subsequent delayed post-infectious neuroimmune involvement reflects evolving immune dysregulation rather than coincident multisystem disease. The patient achieved complete clinical and biochemical recovery on follow-up. Sequential occurrence of AIHA, HLH, and GBS following acute HEV infection within a single hospital admission is rarely described. The case underscores the importance of maintaining a high index of suspicion for evolving extrahepatic immune-mediated complications in patients with acute HEV infection.

## Introduction

Hepatitis E virus (HEV) is a non-enveloped, single-stranded, positive-sense RNA virus belonging to the family Hepeviridae, genus Orthohepevirus. It is transmitted predominantly via the faecal-oral route and remains endemic in regions with inadequate sanitation, including South and Southeast Asia, sub-Saharan Africa, the Middle East, and parts of Mexico, where it is a leading cause of acute viral hepatitis and waterborne outbreaks [1]. The World Health Organization estimates that approximately 20 million HEV infections occur globally each year [2]. HEV encompasses at least eight recognised genotypes; genotypes 1 and 2 are restricted to humans and responsible for epidemic waterborne disease in endemic regions, whereas genotypes 3 and 4 are zoonotic, causing sporadic foodborne infections in industrialised countries, where anti-HEV seroprevalence surveys have reported rates of 10%-52% [3]. In immunocompetent individuals, the infection typically presents as an acute, self-limiting illness characterised by a brief prodrome, jaundice and spontaneous resolution.

While the extrahepatic manifestations of hepatitis B and C virus infections have long been characterised, those associated with HEV have only recently received sustained clinical attention. Neurological complications are the most frequently reported, occurring in approximately 5%-10% of symptomatic cases, and include neuralgic amyotrophy, Guillain-Barré syndrome (GBS), meningoencephalitis, myelitis and peripheral neuropathy [3,4]. Renal involvement - predominantly membranous or membranoproliferative glomerulonephritis and cryoglobulinaemia-associated disease - has been reported in approximately 1%-3% of cases, mainly in chronic HEV infection [3,5]. Haematological abnormalities, including thrombocytopenia, haemolytic anaemia, and, rarely, aplastic anaemia, have been described but remain uncommon [3]. Acute pancreatitis is another infrequent but recognised complication, particularly in South Asian cohorts [3,6]. Musculoskeletal and dermatological involvement are rare and largely confined to isolated reports [3]. The pathogenic basis for these extrahepatic manifestations is multifactorial, encompassing direct extrahepatic viral tropism, dysregulated cytokine-driven immune activation, and molecular mimicry between viral antigens and host tissue antigens [3,4].

A clinically valuable framework for attributing extrahepatic complications to HEV is the triphasic clinical sequence: an initial hepatic phase marked by transaminase elevation and jaundice; a second phase in which the extrahepatic complication emerges - often paradoxically as transaminases normalise, reflecting a transition from direct viral injury to immune-mediated damage; and a third phase of variable recovery or residual deficit [7]. Causality is established by temporal onset of the complication within days to weeks of virologically confirmed HEV infection, positive anti-HEV IgM or detectable HEV RNA at complication onset, and rigorous exclusion of alternative aetiologies [3,4]. Autoimmune haemolytic anaemia (AIHA), when complicating HEV infection, arises through polyclonal B-cell activation and generation of warm autoantibodies against erythrocyte surface antigens; first-line management comprises corticosteroids, with intravenous immunoglobulin (IVIG) or rituximab reserved for refractory disease, and untreated haemolysis risks haemodynamic compromise and thromboembolic events [8]. Secondary haemophagocytic lymphohistiocytosis (HLH) reflects uncontrolled macrophage activation driven by an IFN-γ-dominated cytokine storm; it is managed per the HLH-2004 protocol using dexamethasone and etoposide, and carries mortality exceeding 40% if untreated [9]. GBS arising in the context of HEV infection is attributed to molecular mimicry between viral epitopes and peripheral nerve gangliosides; standard treatment is IVIG or therapeutic plasma exchange, though approximately 20% of patients sustain residual disability at one year and mortality ranges from 3-7% [10].

Although individual extrahepatic complications of HEV are increasingly recognised, the occurrence of multiple immune-mediated complications evolving sequentially in the same patient and during the same episode of acute HEV infection has rarely been documented. We present such a case, in which AIHA, secondary HLH, and GBS arose in temporal sequence during a single admission, and we discuss the implications of this triphasic immune cascade.

## Case presentation

Initial presentation

A 59-year-old man with recently diagnosed type 2 diabetes mellitus presented with progressive fatigue, jaundice and a short preceding febrile illness. He denied alcohol intake for the preceding year, no recent drug exposure suggestive of drug-induced liver injury, or any history of blood transfusion.

On examination, the patient was tachycardic with a pulse rate of 119 beats/min, blood pressure of 94/56 mmHg, and tachypnoeic with a respiratory rate of 23 breaths/min; oxygen saturation was 97% on room air. General examination revealed severe pallor and icterus without lymphadenopathy, pedal oedema, clubbing, cyanosis. Cutaneous examination showed vitiligo patches with no rash, purpura, scratch marks, or tattoo marks. Abdominal examination demonstrated hepatomegaly with the liver palpable 7 cm below the right costal margin and splenomegaly with the spleen palpable 12 cm below the left costal margin along the splenoumbilical line; an umbilical hernia was present, with no evidence of ascites.

Phase I: Autoimmune Haemolytic Anaemia

Laboratory evaluation confirmed acute HEV infection, with anti-HEV IgM positivity and negative serologies for hepatitis A, B, C, and HIV. The peripheral smear demonstrated features of haemolytic anaemia with reticulocytosis (4.92%) and spherocytes. The direct Coombs test was strongly positive (4+), establishing AIHA. Alternative causes of haemolysis, including sickle cell disease, glucose-6-phosphate dehydrogenase deficiency, malaria, dengue, and β-thalassaemia, were excluded. The autoimmune screen, including antinuclear antibody (ANA), anti-smooth muscle antibody (ASMA), anti-liver kidney microsomal antibody (anti-LKM), and perinuclear anti-neutrophil cytoplasmic antibody (p-ANCA), was negative. Abdominal ultrasonography confirmed hepatosplenomegaly without ascites. Initial laboratory findings are detailed in Table 1.

**Table 1:** Initial laboratory investigations at admission. AST, aspartate aminotransferase; ALT, alanine aminotransferase; INR, international normalised ratio; ESR, erythrocyte sedimentation rate; HEV, hepatitis E virus; HBsAg, hepatitis B surface antigen; HCV, hepatitis C virus; HIV, human immunodeficiency virus; HAV, hepatitis A virus; NS1, non-structural protein 1; G6PD, glucose-6-phosphate dehydrogenase; ANA, antinuclear antibody; ASMA, anti-smooth muscle antibody; anti-LKM, anti-liver kidney microsomal antibody; p-ANCA, perinuclear anti-neutrophil cytoplasmic antibody

Investigation	Observed value	Reference range
Haemoglobin	4.3 g/dL	13.0-17.0 g/dL
Total leukocyte count	10,300 cells/µL	4,000-11,000 cells/µL
Platelet count	277,000 cells/µL	150,000-450,000 cells/µL
Reticulocyte count	4.92%	0.5%-2.5%
Peripheral smear	Spherocytes; anisopoikilocytosis	-
Direct Coombs test (DCT)	4+ (positive)	Negative
Indirect Coombs test (ICT)	Positive	Negative
Total bilirubin	22.12 mg/dL	0.2-1.2 mg/dL
Direct bilirubin	10.35 mg/dL	0.0-0.3 mg/dL
Indirect bilirubin	11.77 mg/dL	0.2-0.9 mg/dL
AST (SGOT)	14.6 U/L	10-40 U/L
ALT (SGPT)	14.7 U/L	10-40 U/L
Alkaline phosphatase	64 U/L	40-130 U/L
Gamma-glutamyl transferase	18.1 U/L	8-61 U/L
Serum albumin	2.21 g/dL	3.5-5.0 g/dL
Prothrombin time/INR	14.3 s/1.35	11-13.5 s/0.8-1.1
Urea	175.3 mg/dL	15-40 mg/dL
Creatinine	1.77 mg/dL	0.7-1.3 mg/dL
Sodium	118.5 mmol/L	135-145 mmol/L
Potassium	5.29 mmol/L	3.5-5.0 mmol/L
C-reactive protein	70.97 mg/L	<10 mg/L
ESR	14 mm/hour	0-20 mm/hour
Anti-HEV IgM	Positive	Negative
HBsAg/Anti-HCV/HIV	Negative	Negative
Anti-HAV IgM	Negative	Negative
Dengue NS1/IgM; malaria smear	Negative	Negative
Sickling test; Hb electrophoresis; G6PD assay	Negative/normal	Normal
ANA, ASMA, anti-LKM, p-ANCA	Negative	Negative

Treatment was initiated with intravenous dexamethasone 8 mg once daily, alongside supportive care for acute viral hepatitis.

Phase II: Secondary HLH

Within one week of admission, the patient deteriorated with high-grade fever, hypotension and a toxic appearance despite escalation of broad-spectrum antibiotics. Laboratory studies showed pancytopenia (total leukocyte count 2.26 × 10³/µL with marked thrombocytopenia) and persistent hyperferritinaemia (1,337 ng/mL). Blood and urine cultures were sent and returned negative for any organism. Serum vitamin B₁₂ and folate levels were normal. Bone-marrow aspiration revealed a hypercellular marrow with haemophagocytosis, erythroid hyperplasia, and suppression of myeloid and megakaryocytic lineages. Additional findings included splenomegaly, hypertriglyceridaemia (362 mg/dL) and hypofibrinogenaemia (113 mg/dL).

The patient fulfilled six of the eight HLH-2004 diagnostic criteria, establishing a diagnosis of secondary HLH likely triggered by acute HEV infection and amplified by the preceding immune dysregulation associated with AIHA. Soluble CD25 and NK-cell activity were not assessed. The full criterion-by-criterion assessment is presented in Table 2.

**Table 2:** Assessment against the HLH-2004 diagnostic criteria. Six of eight criteria were fulfilled; two could not be assessed locally. HLH, haemophagocytic lymphohistiocytosis; Hb, haemoglobin; TLC, total leukocyte count; ANC, absolute neutrophil count; NK, natural killer; sIL-2R, soluble interleukin-2 receptor

HLH-2004 criterion	Patient finding	Reference threshold	Status
Fever	Documented fever ≥ 38.3 °C with peaks during the inflammatory phase	Persistent fever ≥ 38.5 °C	Fulfilled
Splenomegaly	Spleen palpable 13 cm below the costal margin	Splenomegaly	Fulfilled
Cytopenias (≥2 lineages)	Hb 6.6 g/dL; platelets 90,000/µL; TLC 2,200/µL	≥2 of: Hb < 9 g/dL, platelets < 100,000/µL, ANC < 1,000/µL	Fulfilled
Hypertriglyceridaemia and/or hypofibrinogenaemia	Triglycerides 362 mg/dL; fibrinogen 113 mg/dL	Triglycerides ≥ 265 mg/dL and/or fibrinogen ≤ 150 mg/dL	Fulfilled
Hyperferritinaemia	Ferritin 1,337 ng/mL	≥500 ng/mL	Fulfilled
Haemophagocytosis	Demonstrated on bone marrow aspirate	Demonstrated in marrow, spleen, lymph node, or liver	Fulfilled
Low/absent NK-cell activity	Not assessed	Below laboratory reference range	Not assessed
Elevated soluble CD25 (sIL-2R)	Not assessed	≥ 2,400 U/mL	Not assessed

HLH-directed therapy was initiated: a single intravenous dose of etoposide 10 mg, dexamethasone escalated from 8 mg once daily to 8 mg twice daily and oral cyclosporin 100 mg once daily. Fever spikes subsequently abated, haematological parameters stabilised, and ferritin levels declined.

Phase III: Guillain-Barré syndrome

**Figure 1:**
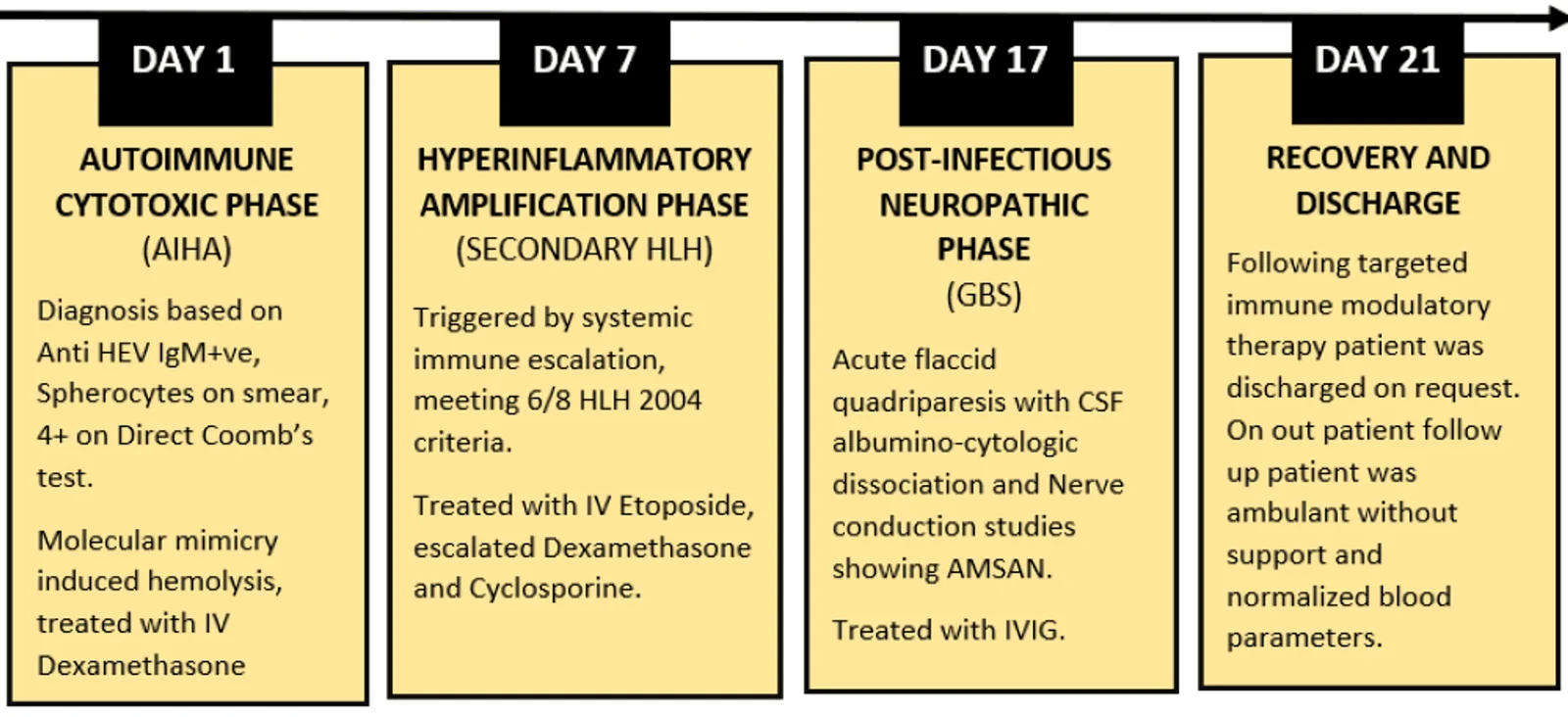
Timeline of events. Image created by the authors using Microsoft Word (Microsoft Corporation, Redmond, WA). AIHA, autoimmune haemolytic anaemia; HLH, haemophagocytic lymphohistiocytosis; GBS, Guillain-Barré syndrome; AMSAN, acute motor sensory axonal neuropathy; IVIG, intravenous immunoglobulin; HEV, hepatitis E virus

On day 17 of admission, the patient developed acute-onset symmetrical quadriparesis with inability to rise from a seated position. Examination revealed flaccid quadriparesis with hypotonia and areflexia in all four limbs, intact cranial nerves, preserved sensorium, and flexor plantar responses. Nerve conduction studies showed an axonal motor-sensory polyneuropathy. Cerebrospinal fluid examination demonstrated albuminocytological dissociation (protein 73 mg/dL; cell count 7/mm³). On the basis of the clinical phenotype, neurophysiology, and CSF findings, an HEV-associated GBS, acute motor sensory axonal neuropathy (AMSAN) variant was diagnosed. Intravenous immunoglobulin 0.4 g/kg/day was administered for five consecutive days (Figure 1).

Hospital course and outcome

By day 21, the patient had improved substantially and was discharged at his request. At discharge, haemoglobin was 9.3 g/dL, total leukocyte count was 1.7 × 10³/µL, platelet count was 60,000/µL, jaundice was resolving, and motor power was MRC grade 3/5 in all four limbs; hepatosplenomegaly persisted. At outpatient follow-up, the patient was ambulant without support. Hepatomegaly had resolved, splenomegaly had markedly regressed, and complete normalisation of haemoglobin, leukocyte count, and platelet count was documented. Total bilirubin had decreased to 1.6 mg/dL, and abdominal ultrasonography demonstrated a normal liver and substantially reduced splenic size.

## Discussion

This case illustrates a striking triphasic immune-mediated evolution following acute HEV infection, characterised sequentially by an autoimmune cytotoxic phase (AIHA), a hyperinflammatory macrophage-activation phase (secondary HLH), and a post-infectious immune-mediated neuropathic phase (GBS). Although HEV has historically been regarded as a hepatotropic, self-limiting virus, accumulating evidence supports a role as a systemic immunological trigger capable of inducing diverse extrahepatic complications and suggests that HEV infection may represent a broader immune-modulating event rather than an isolated hepatic insult [11, 12]. The temporal and immunological progression observed in our patient is consistent with a model of sequential immune dysregulation rather than parallel multisystem involvement. The absence of alternative infectious, autoimmune, malignant, or metabolic aetiologies, together with the stepwise onset of complications following serologically confirmed recent HEV infection, supports a probable temporal association between HEV and the observed immune-mediated manifestations.

Phase I: Autoimmune cytotoxic phase - AIHA

The earliest extrahepatic manifestation in our patient was AIHA, signalling immune dysregulation early in the course of viral infection. Viral infections are well-recognised triggers of autoimmune cytopenias through molecular mimicry, epitope spreading, and bystander activation of autoreactive B cells [13]. In HEV infection, immune-mediated haematological abnormalities including immune thrombocytopenia, haemolytic anaemia, and aplastic anaemia have been described in case reports and small series [14]. The proposed mechanism involves the generation of cross-reactive antibodies directed against erythrocyte membrane antigens following viral antigen presentation. This early cytotoxic phase likely reflects adaptive immune activation directed at viral antigens with unintended autoreactivity, marking the first step in systemic immune deviation. In our patient, AIHA developed following recent HEV infection in the absence of other identifiable triggers for autoimmune haemolysis, favouring HEV-associated immune cytotoxicity.

Phase II: Hyperinflammatory phase - secondary HLH

Following the cytotoxic phase, our patient progressed to a hyperinflammatory state fulfilling HLH diagnostic criteria [9]. Secondary HLH is characterised by uncontrolled macrophage activation driven by excessive cytokine production, particularly interferon-γ, IL-6, and TNF-α. Although Epstein-Barr virus is the most frequently implicated trigger, HEV-associated HLH is increasingly reported [15, 16]. Reported cases describe acute hepatitis followed by cytopenias, hyperferritinaemia, coagulopathy, and multi-organ dysfunction-paralleling the evolution observed in our patient. The transition from antibody-mediated cytopenia to macrophage activation suggests amplification of immune signalling pathways; impaired cytotoxic T-cell and NK-cell function may sustain antigenic stimulation, perpetuating cytokine release and macrophage overactivation [17]. This phase therefore represents an escalation from targeted autoimmunity to systemic immune dysregulation. The development of HLH shortly after the initial haematological manifestation, during the same clinical course following HEV infection, further supports a continuous immune escalation triggered by HEV rather than an independent secondary process.

Phase III: Post-infectious neuropathic phase - GBS

Subsequently, our patient developed GBS, a post-infectious immune-mediated polyradiculoneuropathy. Neurological involvement is among the most frequently reported extrahepatic manifestations of HEV [18]. Both direct viral neurotropism and immune-mediated mechanisms have been proposed, but in many cases HEV RNA is absent from cerebrospinal fluid, favouring a post-infectious autoimmune pathogenesis [19]. Molecular mimicry between viral epitopes and peripheral-nerve gangliosides may produce cross-reactive antibodies that mediate demyelination or axonal injury. The delayed onset of GBS following systemic hyperinflammation in our patient is more consistent with a post-infectious immune-reconstitution phenomenon than with direct viral invasion. The temporal appearance of GBS during recovery from the systemic inflammatory phase, without any other identifiable antecedent infectious trigger, further strengthens the likelihood of HEV-associated post-infectious neuroimmune dysregulation.

Multisystem extrahepatic involvement in HEV: literature context

Several reports have documented simultaneous or sequential involvement of multiple extrahepatic systems in individual HEV-infected patients. Comprehensive reviews by Kamar et al. have described the neurological, renal, and haematological complications associated with HEV infection, supporting the concept of systemic immune activation beyond hepatic injury [3,12,18]. Discrete reports have separately documented HEV-associated HLH with concurrent cytopenias and systemic inflammation [15], and HEV-associated GBS, reinforcing the virus's neuroimmunological potential [19,20]. However, documentation of a clearly sequential triphasic immune progression encompassing autoimmune cytopenia, HLH, and post-infectious neuropathy in a single patient remains exceedingly uncommon.

Proposed model of sequential immune escalation

The immunological trajectory in this case may be conceptualised as a continuum rather than discrete events: an initial autoimmune activation phase (localised cytotoxic immune response with autoantibody formation), followed by a hyperinflammatory amplification phase (macrophage activation and cytokine storm), and finally a post-infectious immune-reconstitution phase (delayed antibody-mediated neuropathy). HEV infection may therefore serve as the initial trigger in a genetically or immunologically predisposed host, with subsequent escalation driven by dysregulated cytokine networks. Rather than independent complications, these manifestations may represent different expressions of a unified immune cascade.

Gap in the literature and clinical implications

Although extrahepatic manifestations of HEV are increasingly reported, most publications describe isolated complications-autoimmune cytopenia, HLH, or neurological involvement-rather than a temporally ordered multiphasic immune progression. There remains limited literature characterising sequential immune escalation in acute HEV infection, the transition from antibody-mediated cytotoxicity to macrophage activation, the relationship between hyperinflammation and subsequent post-infectious neuropathy, and predictors of progression from uncomplicated hepatitis to systemic immune dysregulation. Recognition of such phased immune progression has practical importance, since early identification of immune amplification may permit timely immunomodulatory intervention and improve outcomes.

## Conclusions

This case illustrates a rare presentation of acute HEV infection manifesting as phased immune dysregulation with sequential multisystem involvement. The clinical course evolved through three distinct immune-mediated phases: an autoimmune cytotoxic phase (AIHA), a hyperinflammatory macrophage-activation phase (secondary HLH), and a post-infectious immune-mediated neuropathic phase (GBS). This progression underscores the capacity of HEV to drive dynamic and escalating immune dysregulation beyond hepatic injury. Early recognition of evolving systemic complications and timely initiation of appropriate immunomodulatory therapy were pivotal in achieving complete clinical and haematological recovery. A high index of suspicion for systemic immune-mediated complications is warranted in acute HEV infection whenever unexplained haematological or neurological abnormalities arise.

## References

[1] World Health Organization (2026). Hepatitis E. https://www.who.int/news-room/fact-sheets/detail/hepatitis-e.

[2] Centers for Disease Control and Prevention (2026). Hepatitis E Questions and Answers for Health Professionals. https://www.cdc.gov/hepatitis/hev/index.htm.

[3] Kamar N, Dalton HR, Abravanel F, Izopet J (2014). Hepatitis E virus infection. Clin Microbiol Rev.

[4] Pischke S, Hartl J, Pas SD, Lohse AW, Jacobs BC, Van der Eijk AA (2017). Hepatitis E virus: infection beyond the liver?. J Hepatol.

[5] Kovvuru K, Carbajal N, Pakanati AR (2022). Renal manifestations of hepatitis E among immunocompetent and solid organ transplant recipients. World J Hepatol.

[6] Nayak HK, Kamble NL, Raizada N, Garg S, Daga MK (2013). Acute pancreatitis complicating acute hepatitis e virus infection: a case report and review. Case Reports Hepatol.

[7] van Eijk JJ, Dalton HR, Ripellino P (2017). Clinical phenotype and outcome of hepatitis E virus-associated neuralgic amyotrophy. Neurology.

[8] Jäger U, Barcellini W, Broome CM (2020). Diagnosis and treatment of autoimmune hemolytic anemia in adults: recommendations from the First International Consensus Meeting. Blood Rev.

[9] Henter JI, Horne A, Aricó M (2007). HLH- 2004: diagnostic and therapeutic guidelines for haemophagocytic lymphohistiocytosis. Pediatr Blood Cancer.

[10] van den Berg B, Walgaard C, Drenthen J, Fokke C, Jacobs BC, van Doorn PA (2014). Guillain-Barré syndrome: pathogenesis, diagnosis, treatment and prognosis. Nat Rev Neurol.

[11] Dalton HR, Kamar N, Baylis SA, Moradpour D, Wedemeyer H, Negro F (2018). EASL Clinical Practice Guidelines on hepatitis E virus infection. J Hepatol.

[12] Kamar N, Bendall R, Legrand-Abravanel F, Xia NS, Ijaz S, Izopet J, Dalton HR (2012). Hepatitis E. Lancet.

[13] Cusick MF, Libbey JE, Fujinami RS (2012). Molecular mimicry as a mechanism of autoimmune disease. Clin Rev Allergy Immunol.

[14] Woolson KL, Forbes A, Vine L (2014). Extra-hepatic manifestations of autochthonous hepatitis E infection. Aliment Pharmacol Ther.

[15] Desai N, Vetal D, Kulkarni A, Karnik N, Trivedi T (2024). Hemophagocytic lymphohistiocytosis: a rare complication of acute hepatitis E infection. J Assoc Physicians India.

[16] Péron JM, Bureau C, Poirson H (2007). Fulminant liver failure from acute autochthonous hepatitis E in France: description of seven patients with acute hepatitis E and encephalopathy. J Viral Hepat.

[17] Janka GE (2007). Hemophagocytic syndromes. Blood Rev.

[18] Kamar N, Bendall RP, Peron JM (2011). Hepatitis E virus and neurologic disorders. Emerg Infect Dis.

[19] Dalton HR, Kamar N, van Eijk JJ, Mclean BN, Cintas P, Bendall RP, Jacobs BC (2016). Hepatitis E virus and neurological injury. Nat Rev Neurol.

[20] Aggarwal R (2011). Clinical presentation of hepatitis E. Virus Res.

